# The prognostic role of EZH2 expression in rectal cancer patients treated with neoadjuvant chemoradiotherapy

**DOI:** 10.1186/1748-717X-9-188

**Published:** 2014-08-27

**Authors:** Xiangjiao Meng, Zhaoqin Huang, Renben Wang, Yuhong Jiao, Huijuan Li, Xiaoqing Xu, Rui Feng, Kunli Zhu, Shumei Jiang, Hongjiang Yan, Jinming Yu

**Affiliations:** Department of Radiation Oncology of Shandong Cancer Hospital and Institute, No. 440 Jiyan Road, Jinan, Shandong 250117 China; Department of Radiology, Provincial Hospital Affiliated to Shandong University, Jinan, Shandong 250021 China; Department of Medicine of Shandong Cancer Hospital and Institute, Jinan, Shandong 250117 China

**Keywords:** Rectal cancer, EZH2, Neoadjuvant chemoradiotherapy, Tumor response, Prognosis

## Abstract

**Background:**

Neoadjuvant chemoradiotherapy (nCRT) combined with surgery has been implemented as a standard treatment strategy in locally advanced rectal cancer (LARC). However, there is a wide spectrum of response to nCRT. The aim of this study was to determine whether enhancer of zeste homologue 2 (EZH2 ) expression could predict response to nCRT and outcomes for patients in LARC.

**Method:**

The study examined the EZH2 expression in 112 biopsies by immohistochemistry. The associations between EZH2 and clinical characters were analyzed.

**Results:**

EZH2 expression in biopsy tissue was significantly related to increased tumor cell proliferation, as assessed by Ki-67 expression with a cutoff value of 37% (p <0.001). High EZH2 expression was correlated closely with low differentiation (p = 0.029), high CEA level (p = 0.041), T4 status (p = 0.011) and node metastasis (p =0.045). By univariate and multivariate analysis, we observed low EZH2 expression could reliably and independently predict the good response to nCRT ( p = 0.026 and p = 0.023) and down-staging ( p = 0.021 and p = 0.027). In univariate analysis, high EZH2 expression was significantly associated with poor 5-year disease-free survival (p = 0.025) and 5-year overall survival (p = 0.032). In multivariate analysis, EZH2 was a prognostic factor for 5-year DFS (HR = 2.287; 95% CI 1.137-4.602, p = 0.020) but not for 5-year OS (HR = 2.182; 95% CI 0.940-5.364, p = 0.069).

**Conclusion:**

Our study revealed that low EZH2 expression in biopsy tissue might be a useful predictive factor of good tumor response to nCRT and longer 5-year DFS in patients with LARC. However this is a relatively small retrospective study, to further validate the role of EZH2 in rectal cancer, large consistent cohort studies are needed.

## Background

Rectal cancer is one of the leading causes of cancer-related mortality and morbidity in the world [1]. Over the last two decades, advances in new treatment strategies have contributed significantly to the improvement of local recurrence and overall survival rate in patients with locally advanced rectal cancer (LARC) [2, 3]. Evidences from randomized clinical trials strongly support the treatment of rectal cancer with neoadjuvant chemoradiotherapy (nCRT) and surgery [4, 5]. Due to the heterogeneous responses to nCRT, identifying a predictive or prognostic molecular marker is important to discriminate those patients who would benefit from nCRT and avoid unnecessary treatment with toxic side effects.

Enhancer of zeste homologue 2 (EZH2), as a member of the polycomb group of genes has important functions in tumor aggressiveness [6]. Its amplification was first studied in hematologic malignancies [7]. Disruption of EZH2 expression was observed to correlate closely with tumor aggressiveness and/or poor patient prognosis in prostate [8], oral [9], bladder [10], esophageal [11], breast [12] and ovarian cancers [13]. EZH2 is a cell cycle regulator and the expression of EZH2 delays upon tissue maturation and differentiation [14]. Over-expression of EZH2 was also observed to shorten the G1 phase of the cell cycle and lead to accumulation of cells in the S phase [15], which might play a critical role in radioresistance [11, 16]. Furthermore, as an essential downstream target of pRB/E2F pathway, EZH2 is a critical mediator of E2F function [15]. E2F1 has been observed to be associated with radiosensitivity and/or chemosensitivity in certain types of tumors [17–19]. In esophageal squamous cell carcinoma (ESCC), high EZH2 expression was significantly correlated with lack of clinical complete response to CRT (p = 0.028) and poor disease-specific survival (p < 0.001) [11]. In atypical teratoid/rhabdoid tumor (ATRT), targeted disruption of EZH2 by RNAi or pharmacologic inhibition strongly impaired cell growth, suppressed cell self-renewal, induced apoptosis, and potently sensitized these cells to radiation [16].

However, there is limited study concentrating on the predicting role of EZH2 in LARC. In the present study we examined the expression of EZH2 protein in pretreatment biopsies by immunohistochemistry (IHC), so as to determine whether EZH2 expression has predictive value of nCRT response and prognosis in patients with LARC.

## Materials and methods

### Patients and clinical assessment

The clinicopathological characteristics were reviewed retrospectively for the purpose of the study. All patients were diagnosed with primary rectal adenocarcinoma confirmed by rigid rectoscopy. Before treatment, tumor-node-metastasis (TNM) stage was determined by a series of examination including physical examination, carcinoembryonic antigen (CEA) serum level, chest computed tomography (CT), contrast-enhanced CT and/or magnetic resonance imaging (MRI) of the abdomen and pelvis. TNM stages were reported according to the American Joint Committee on Cancer (AJCC) [20]. Informed consents were obtained from all patients and the research protocols were approved by the Ethics Committee of Shandong Cancer Hospital and Institute.

### Multimodal treatment

112 patients received concurrent chemotherapy and radiotherapy followed by surgery. In brief, patients underwent whole pelvis preoperative radiotherapy with a dose of 50.4 Gy in 28 fractions using three-dimensional conformal irradiation or four-field box technique. Concurrent chemotherapy regimens include continuous infusion of 5-fluorouracil ± oxaliplatin or capecitabine ± oxaliplatin. Four to six weeks after the completion of nCRT, total mesorectal excision (TME) was performed.

### Pathologic assessment

Histopathologic examination was performed by two pathologists who were blinded for all clinical data. Pathologic TNM staging was performed on the surgical specimens to assess for tumor down-staging according to the current classification [20].

Tumor response was also evaluated using the tumor regression grade (TRG) system proposed by Dworak et al. [21]. Details as follows: grade 0, no regression; grade 1, minor regression; grade 2, moderate regression; grade 3, good regression; and grade 4, total regression, no viable tumor cells. In the present study, TRG 3 and 4 were defined as “good response” while TRG 0–2 were defined as “poor response.”

### Immunohistochemistry

Tumor samples were collected from pretreatment tumor biopsies in 112 patients who received nCRT. The EZH2 and Ki-67 status was assessed using paraffin-embedded tissue samples that were cut into 5 μm slices. The process of staining was performed according to the product protocol. Briefly, all sections were deparaffinized in xylene and rehydrated with distilled water through a graded series of ethanol solutions. Antigen retrieval was performed under high pressure for 2 minutes. Nonspecific binding was blocked by the application of serum at 37°C for 15 minutes (Beijing Zhongshan Golden Bridge Biotechnology Company, Beijing, China). For EZH2, the sections were stained with primary monoclonal rabbit anti-human EZH2 antibody ( Abcam, Cambridge, UK) in humidified chamber at 37°C for 60 minutes, with a diluted ratio of 1:200. Secondary goat anti-rabbit antibody was incubated at 37°C for 30 minutes (Beijing Zhongshan Golden Bridge Biotechnology Company, Beijing, China). For Ki-67, the sections were stained with primary monoclonal mouse anti-human Ki-67 antibody (Beijing Zhongshan Golden Bridge Biotechnology Company, China) in humidified chamber at 37°C for 60 minutes, with a diluted ratio of 1:100. Secondary goat anti-mouse antibody was incubated at 37°C for 30 minutes. Both EZH2 and Ki-67 expression were visualized using 3,3’-diaminobenzidine (DAB) and subsequently counterstained with hematoxylin.

Two independent observers who were blinded to the clinicopathologic parameters performed scoring using a previously validated scoring system for EZH2 expression [10, 11, 22, 23]. This system scores nuclear EZH2 expression by recording the percentage of positive nuclei staining for the EZH2 protein, irrespective of staining intensity. EZH2 staining was classified into 2 groups: high expression, when at least 50% of the cells showed positive immunoreactivity in the nuclei and low expression, when positive cells were less than 50%. For Ki-67 immunohistochemistry, the percentage of positive nuclei staining tumor cells was assessed in five representative visual fields. A cutoff was then chosen at the median value, dividing the samples in low or high nuclear Ki-67 expression.

### Follow-Up

Patients underwent a standardized post-treatment follow-up including physical examinations, CEA serum level, peripheral blood cell count, chest X-ray, every 3 months for the first 2 years and every 6 months thereafter. Patients also underwent abdominal and pelvic CT or MRI every 6 months. Colonoscopy was performed within 1 year after treatment and then once every 2–3 years. The median follow-up was 56 months.

### Statistical analysis

For all the statistical analysis, Statistical Product and Service Solutions (SPSS17.0) was used. The chi-squared test was also performed to assess the association between pathologic factors including EZH2 expression and tumor response. A multivariate stepwise logistic regression analysis was performed in order to determine the independent prediction of all variables that were significant in the univariate analysis. The associations between EZH2 protein expression and Ki-67 was also analyzed by Chi-square test. For survival analysis, overall survival (OS) was defined as the time from diagnosis to death from any causes and was censored at the date of last contact for surviving patients. Disease free survival (DFS) was defined as the time from diagnosis to any evidence of local or systemic cancer recurrence. The impact of the EZH2 status on OS and DFS was determined using Kaplan-Meier method for the univariate survival analysis and Cox proportional hazards model for the multivariate survival analysis. The p values < 0.05 was considered statistically significant differences.

## Results

### Patient characteristics

The clinicopathological parameters of the 112 patients was detailed in Table 1. There were 62 cases assessed as clinical T3 (cT3) while 50 cases as clinical T4 (cT4) and 38 patients assessed as clinical N0 (cN0) while 74 patients assessed as cN+. There were 52 cases with CEA < 3.4 ng/ml and 60 cases with CEA ≥ 3.4 ng/ml. With respect of tumor localization, 39 tumors were localized in <6 cm and 73 localized in >6 cm from the anal verge. There were 44 cases assessed as pathological T1-2 (pT1-2) while 68 cases as pathological T3-4 (pT3-4) and 67 patients assessed as stage pathological N0 (pN0) while 45 patients assessed as pathological N + (pN+). A comparison of pretreatment staging results and histopathologic diagnosis after surgery revealed AJCC down-staging in 52.7% patients (n = 59). Tumor regression grade analysis showed good response (TRG 3 and TRG4) in 47.3% patients (n = 53).

**Table 1:** **Correlations between EZH 2 expression and clinicopathological parameters**

Clinicopathological parameters	Cases n = 112	EZH 2 expression		***p***
		Low	High	value
Gender				
Male	71	32	39	0.530
Female	41	21	20	
Age(years)				
<62	49	26	23	0.283
≥62	63	27	36	
Histology				
Differentiated	64	36	28	0.029
Undifferentiated	48	17	31	
CEA(ng/ml)				
<3.4	52	30	22	0.041
≥3.4	60	23	37	
Distance from anal verge (cm)				
<6	39	20	19	0.539
≥6	73	33	40	
Clinical Tumor status				
cT3	62	36	26	0.011
cT4	50	17	33	
Clinical Node status				
cN0	38	23	15	0.045
cN+	74	30	44	
Ki-67				
Low	56	42	14	<0.001
High	56	11	45	

### Expression of EZH2 in LARC

**Figure 1:**
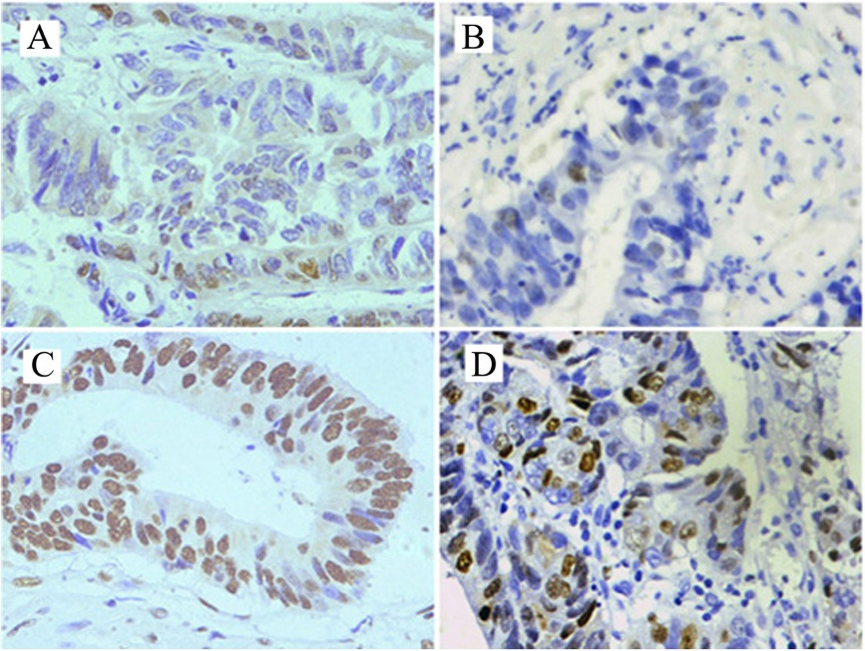
**Immunohistochemical staining for EZH2 and Ki-67 in rectal cancer cell nucleus. A**. Low expression of EZH2 (400×). **B**. Low expression of Ki-67 (400×). **C**. High expression of EZH2 (400×). **D**. High expression of Ki-67 (400×).

In this study, protein expression of EZH2 was examined by IHC(Figure 1). High expression of EZH2 was observed in 59/112 (52.7%) biopsy specimens from patients underwent nCRT. The median Ki-67 expression is 37%. The association between clinicopathological parameters and EZH2 expression levels were summarized in Table 1. EZH2 expression was significantly related to increased tumor cell proliferation, as assessed by Ki-67 expression with a cutoff value of 37% (*p* <0.001).High EZH2 expression was correlated closely with low differentiation (p = 0.029), high CEA level (*p* = 0.041), T4 status (p = 0.011) and Node metastasis (p =0.045).

### Correlation between EZH2 expression and CRT response

To determine the ability of clinicopathological parameters to predict good histopathologic tumor response and down-staging to nCRT, the chi-square test was used (Table 2). It seemed that EZH2 low expression could predict the response to nCRT: 30 cases in 53 EZH2 low expression patients achieved good tumor response while 21 cases in 59 EZH2 high expression patients did (p = 0.026); 34 cases in 53 EZH2 low expression patients achieved down-staging while 25 cases in 59 high expression patients did (p = 0.021). Low CEA level and non node metastasis also showed significant correlation with good tumor regression (p = 0.016 and p = 0.022) and down-staging (p = 0.033 and p = 0.046). Tumors with cT3 were more likely to achieve good response than those with cT4 (p = 0.01).

**Table 2:** **Correlations between clinicopathological parameters and tumor response**

Parameters	Cases N = 112	Tumor regression grade		***p***value	Tumor		***p***value
		Good response	Poor response		Down-staging	Non-down-staging	
Age							
<62	49	24	25	0.519	26	23	0.943
≥62	63	27	36		33	30	
Sex							
Male	71	34	37	0.511	41	30	0.157
Female	41	17	24		18	23	
Distance from anal verge (cm)							
<6	39	18	21	0.924	23	16	0.329
≥6	73	33	40		36	37	
Histology							
Differentiated	64	28	36	0.661	31	33	0.299
Undifferentiated	48	23	25		28	20	
CEA(ng/ml)							
<3.4	52	30	22	0.016	33	19	0.033
≥3.4	60	21	39		26	34	
Clinical Tumor status							
cT3	62	35	27	0.01	37	25	0.099
cT4	50	16	34		22	28	
Clinical Node status							
cN0	38	23	15	0.022	25	13	0.046
cN+	74	28	46		34	40	
EZH2							
Low	53	30	23	0.026	34	19	0.021
High	59	21	38		25	34	

By multivariate stepwise logistic regression analysis, we found low expression of EZH2 showed significant correlation with good tumor regression (OR = 2.684; 95% CI 1.147-6.280, p = 0.023) and down staging (OR = 2.476; 95% CI 1.107-5.537, p = 0.027). Moreover, cN was also kept in the model as a predictive factor for good tumor response (p = 0.018) and down-staging (p = 0.025) (Table 3).

**Table 3:** **Multivariate analysis for tumor response**

Parameters	Odds ratio	95% confidence interval	P value
Good response			
CEA	1.940	0.822-4.581	0.130
cT	2.580	1.080-6.161	0.033
cN	2.869	1.199-6.864	0.018
EZH2	2.684	1.147-6.280	0.023
Down-staging			
CEA	1.624	0.735-3.589	0.231
cN	2.915	1.239-6.860	0.025
EZH2	2.476	1.107-5.537	0.027

EZH2: Enhancer of zeste homologue 2; CEA: carcinoembryonic antigen.

### Association of EZH 2 expression with Survival

**Figure 2:**
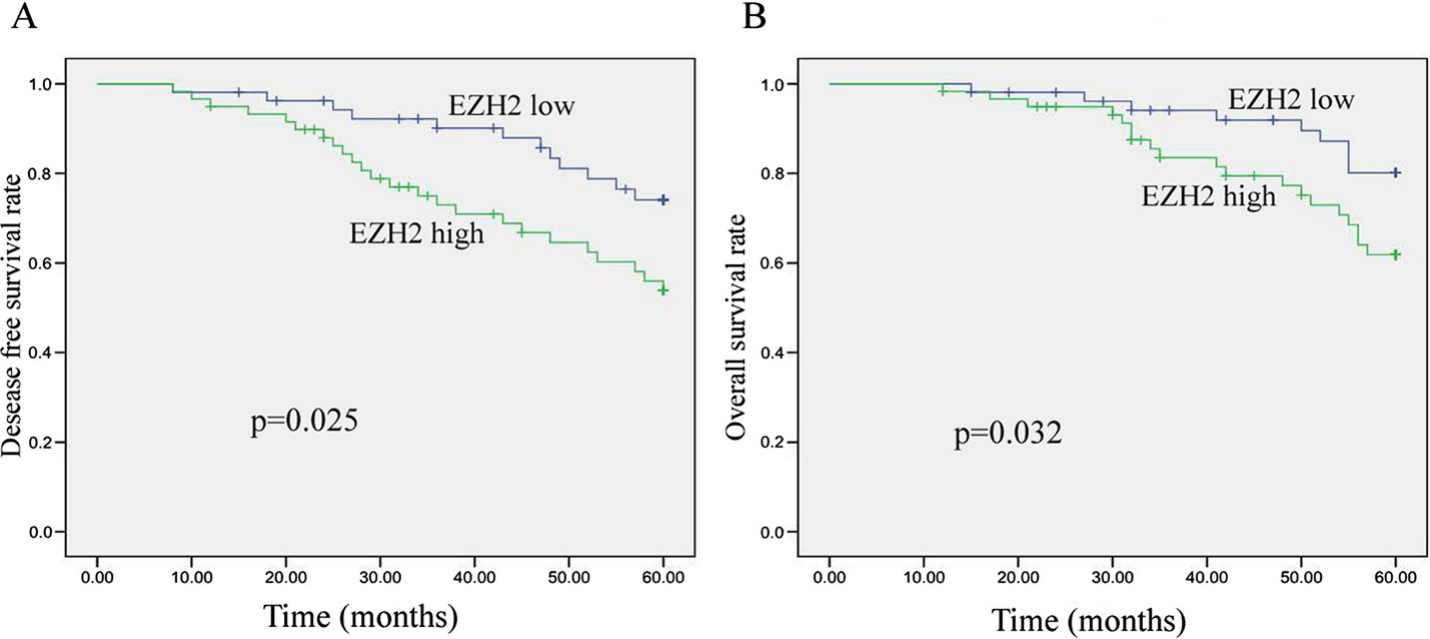
**Kaplan-Meier estimates of disease-free survival(DFS) and overall survival (OS) rates in relation to EZH2 expression. A**. The 5-year DFS was significantly shorter in patients with high expression patients than in patients with low expression(*p* = 0.025). **B**. The 5-year OS was significantly shorter in patients with high expression patients than in patients with low expression (*p* = 0.032).

EZH2 status, cN, cT, pathological tumor status (pT) and pathological node status (pN) ,were found to be significantly correlated with 5-year DFS and OS in univariate analysis. As listed in Table 4, among 112 patients who underwent nCRT, the 5-year DFS of 59 patients with EZH2 high expression was significantly poorer than that of 53 patients with EZH2 low expression (p = 0.025) (Figure 2A). In the total study population, EZH2 high expression patients had significant poorer 5-year OS as compared with EZH2 low expression patients (p = 0.032) (Figure 2B). In multivariate analysis, we found the pN was an important prognostic factor both for two end points while the pT was only the prognostic factor of 5-year DFS (Table 5). EZH2 was a prognostic factor for 5-year DFS (HR = 2.287; 95% CI 1.137-4.602, p = 0.020) but not for 5-year OS (HR = 2.182; 95% CI 0.940-5.364, p = 0.069) (Table 5).

**Table 4:** **Univariate analysis between clinicopathological parameters and survival**

Parameters	Cases	5-year DFS (%)	***P***value	5-year OS(%)	***P***value
Gender					
Male	71	62.8	0.631	76.7	0.354
Female	41	64.4		67.4	
Age					
<62	49	64.2	0.615	69.9	0.476
≥62	63	62.7		76.4	
Distance from anal verge (cm)					
<6	39	59.9	0.375	68.4	0.286
≥6	73	66.1		76.7	
Histology					
Differentiated	64	68.0	0.267	78.2	0.139
Undifferentiated	48	56.3		66.5	
CEA (ng/ml)					
<3.4	52	68.2	0.320	77.0	0.380
≥3.4	60	58.9		66.7	
Clinical Tumor status					
cT3	62	70.0	0.046	81.4	0.021
cT4	50	54.2		59.4	
Pathological Tumor status					
pT0-2	44	73.3	0.034	83.4	0.019
pT3-4	68	56.8		65.3	
Clinical Node status					
cN0	38	78.6	0.011	84.0	0.008
cN+	74	51.9		59.5	
Pathological Node status					
pN0	67	69.4	0.016	79.9	0.024
pN+	45	54.5		61.7	
EZH 2					
High	53	74.0	0.025	82.8	0.032
Low	59	53.8		64.1	

DFS: Disease free survival; OS:overall survival.

**Table 5:** **Multivariate analysis for survival**

Parameters	Hazard ratio	95% confidence interval	P value
5-year-DFS			
EZH2	2.287	1.137-4.602	0.020
pT	2.244	1.076-4.678	0.013
pN	2.532	1.292-4.961	0.007
5-year-OS			
EZH2	2.182	0.940-5.064	0.069
pT	2.395	0.997-5.365	0.051
pN	2.471	1.138-5.364	0.022

## Discussion

In this study, we first examined the expression of EZH2 in LARC and its predicting significance in 112 patients treated with nCRT. Our results showed that the high expression of EZH2 correlated with undifferentiation, high CEA level, T4 status and lymph node metastasis status at diagnosis. By univariate and multivariate analysis, we observed low expression of EZH2 showed significant correlation with good tumor regression (p = 0.026) and down-staging (p = 0.021), which means EZH2 can reliably and independently predict the response to nCRT with LARC.

The results from the current study are in accordance with the previous studies in other solid cancers [11, 17]. In esophageal squamous cell carcinoma (ESCC), high EZH2 expression was significantly correlated with increased cell proliferation (p = 0.009) and lack of clinical complete response to CRT (p = 0.028) [11]. Pharmacologic inhibition of EZH2 suppressed cell growth and increased radiation sensitivity of ATRT cells [16]. In lung cancer, EZH2 silencing with RNA interference (RNAi) enhances A549 and HTB-56 cell sensitivity to irradiation both in vitro and in vivo [23].

The sensitivity of chemotherapy and/or radiotherapy is a complex phenomenon and regulated by a series of internal and extrinsic factors, including cell cycle arrest, cell apoptosis, and DNA damage repair [24]. There are two mechanisms possibly associated with resistance to CRT of high EZH2 expression. First, EZH2 is a cell cycle regulator [14]. A cDNA microarray study demonstrated that disruption of EZH2 expression can retard cell proliferation [25]. It is a fact that cells are not equally sensitive to radiation throughout the cell cycle but show increased radiation sensitivity in G2/M phase and decreased radiation sensitivity in S phase. Over-expression of EZH2 was observed to shorten the G1 phase of the cell cycle and lead to accumulation of cells in the S phase [15]. Inhibition of EZH2 by interfering RNA (RNAi) led to significant inhibition of DNA synthesis and increased the percentage of ATRT cells in G2/M phase [16]. EZH2 might be responsible, at least in part, for the tumor resistance to radiotherapy [11]. Second, as an essential downstream target of pRB/E2F pathway, EZH2 is a critical mediator of E2F function [15]. E2F1 has been observed to be associated with radiosensitivity and/or chemosensitivity in certain types of tumors [17–19]. Thus, the pRB/E2F/EZH2 pathway may be one of the mechanisms involved in tumor response to CRT.

EZH2 high expression has been studied in many solid tumors [8–13]. But up to now only one report studied EZH2 expression in 409 colorectal cancer patients but found no association between EZH2 expression and prognosis in rectal cancer [26]. In this study we first showed the significant correlation between EZH2 high expression and poor prognosis in LARC. The differences can be explained in two aspects: First, the above study used the resected tissue while our study used biopsy tissues. Second, the patients in the above study took surgery directly while the patients of this study received concurrent chemotherapy and radiotherapy followed by surgery.

In our study, EZH2 over-expression could be a valuable prognostic indicator of poor 5-year DFS both in univariate and multivariate analyses. Refers to 5-year OS, in univariate analysis, EZH2 over-expression could predict poor outcome but there is no significant difference in multivariate analysis. Although the difference was not significantly, it still revealed a trend the EZH2 over expression showed shorter 5-year OS (p = 0.069).

The similar results were also observed in other solid tumors [8–13]. In metastatic prostate cancer, EZH2 upregulated oncogenes and loss of EZH2 inhibited the proliferation of cancer cells [8, 19]. In breast cancer, abnormally elevated EZH2 levels have been found to be highly correlated with tumor cell invasiveness and increased proliferation rates, poor prognosis [27, 28]. In ESCC, high expression of EZH2 was also observed to be an independent prognostic factor [11].

The possible mechanisms underlying associations of high expression of EZH2 and the poor prognosis is very complexed. The functional consequence of increased EZH2 expression in cancer tissues includes the silencing of genes that promote differentiation and restrain proliferation [29]. Currently, EZH2 has been found to function as a transcriptional repressor that silences an array of target genes, including more than 200 tumor suppressors [30]. In addition to its role as a transcriptional repressor, several studies have shown that EZH2 may also function in target gene activation [31–33]. Furthermore, overexpression of EZH2 is involved in many signaling pathways, such as the pRB-E2F, PI3K/Akt, estrogen receptor and c-Myc signal transduction pathways [8, 15, 25, 27, 34]. These results collectively, suggest that EZH2 high expression were significantly associated with tumor invasiveness and poor prognosis, the regulation of protein expression of EZH2 is quite complicated, and it may be a promising candidate for anti-cancer treatment.

## Conclusion

In this study, we observed, for the first time, EZH2 expression in LARC could predict the response to nCRT. EZH2 high expression were significantly associated with shorter 5-year disease-free survival and 5-year overall survival in univariate analysis. However, some inherent limitations of this study might lead to biased results. First, the current study was a retrospective study and the number of patients in our study was limited. Second, the chemotherapeutic regimens were various, which could affect the tumor response to nCRT [35]. Third, for tumor heterogeneity, weather a biopsy is representative of the whole tumor need to be investigated. To further validate the role of EZH2 in rectal cancer, large consistent cohort studies are needed.

## Abbreviations

LARC: Locally advanced rectal cancer
nCRT: Neoadjvant chemoradiotherapy
EZH2: Enhancer of zeste homologue 2
IHC: Immunohistochemistry
EGFR: Epidermal growth factor receptor
VEGF: Vascular endothelial growth factor
TNM: Tumor-node-metastasis
CEA: Carcinoembryonic antigen
CT: Computed tomography
MRI: Magnetic resonance imaging
AJCC: American Joint Committee on Cancer
TME: Total mesorectal excision
TRG: Tumor regression grade
DAB: 3,3’-diaminobenzidine
OS: Overall survival
DFS: Disease free survival
SPSS: Statistical Product and Service Solutions
ESCC: Esophageal squamous cell carcinoma
ATRT: Atypical rhabdoid teratoid tumor
cT: Clinical tumor
cN: Clinical node status
pT: Pathological tumor
pN: Pathological node status.
